# Body composition assessment in 6-month-old infants: A comparison of two- and three-compartment models using data from the Baby-bod study

**DOI:** 10.1038/s41430-023-01394-5

**Published:** 2024-01-17

**Authors:** Manoja P. Herath, Jeffrey M. Beckett, Sisitha Jayasinghe, Nuala M. Byrne, Kiran D. K. Ahuja, Andrew P. Hills

**Affiliations:** School of Health Sciences, College of Health and Medicine, University of Tasmania, Launceston, TAS 7248 Australia

**Keywords:** Paediatrics, Clinical trials

## Abstract

**Background/Objectives:**

An appreciation of infant body composition is helpful to understand the ‘quality’ of growth in early life. Air displacement plethysmography (ADP) using PEA POD and the deuterium dilution (DD) technique are commonly used body composition approaches in infants. We evaluated the comparability of body composition assessed using both techniques with two-compartment (2C) and three-compartment (3C) models in 6-month-old infants.

**Subjects/Methods:**

Infant fat mass (FM) and percent fat mass (%FM) obtained from a 2C model using PEA POD (2C-PP) and a 2C model using the deuterium dilution technique (2C-DD) were compared to those derived from a 3C model, and to each other, using Bland-Altman analysis and Deming regression.

**Results:**

Measurements were available from 68 infants (93% Caucasian, 53% male). The mean biases were not significant between any of the method comparisons. However, significant constant and proportional biases were identified in 2C-DD vs 3C and 2C-PP vs 2C-DD, but not in the 2C-PP vs 3C comparison. Furthermore, we observed significant associations between the mean differences and infants’ percent total body water (%TBW).

**Conclusions:**

While no significant between-method mean differences were found in body composition estimates, some comparisons revealed significant constant and proportional biases and notable associations between the mean differences and %TBW were observed. Our results emphasise the importance of method choice, ensuring methodological uniformity in long-term studies, and carefully considering and regulating multiple pre-analytical variables, such as the hydration status of the participants.

## Introduction

Assessment of infant body composition has become an increasingly important area of research due to the association between early growth and subsequent risk of obesity and metabolic diseases [1–3]. Body composition can be assessed using a range of approaches, with most common methods dividing the body into two compartments (2C): fat mass (FM) and fat-free mass (FFM), and assuming that the densities of FM and FFM remain constant between individuals [4]. However, while the density of FM remains stable across the lifespan, the density of FFM varies by age and other individual factors, such as whole-body hydration status, with the highest values found in infancy [5, 6]. The 3-compartment (3C) model divides body mass into FM, total body water (TBW), and fat-free dry mass (FFDM), and produces more valid body composition results than the 2C model as it accounts for the inter-individual variation of TBW. The 4-compartment (4C) model creates a fourth component by dividing the FFDM component in the 3C model into proteins and minerals [7]. Despite being the “gold standard reference method”, 4C models require measurements using various techniques, which makes the model more expensive and increases the participant burden. Therefore, 2C and 3C models are considered more suitable approaches in clinical or field studies, including with infants.

Despite the availability of various body composition techniques, using them in the paediatric population can be associated with a range of practical challenges due to the distinctive physiological and behavioural characteristics of infants. For example, the use of dual-energy X-ray absorptiometry (DXA) raises concerns regarding radiation exposure (albeit being extremely low), and keeping an infant motionless for a DXA scan can be problematic [8]. Air displacement plethysmography (ADP) using the PEA POD to estimate body density is arguably one of the most “practical” approaches for body composition assessment during early infancy [9]. The technique has several advantages, including ease of use, a very short assessment time, being non-invasive, and not being affected by an infant’s behavioural state (e.g., movement, crying, urination). The technique also has good precision; however, it is limited to assessment of infants up to ~6 months of age (a body mass of 8-10 kg) [10]. The adult ADP system (BOD POD) adapted with a paediatric option has demonstrated good validity with the 4C model in 2–6 years old children [11]; however, to date, there has been a gap in using ADP technology for 6–24 months old infants. The deuterium dilution (DD) technique, which estimates TBW, is another commonly used body composition approach in infancy (and suited to all ages). The DD technique has several advantages, including safety, suitability for field use, and collected samples can be stored for extended periods prior to analysis [8]. The practical challenges involved with the use of the DD technique in infants include dose losses due to spills and extended waiting periods between sample collections [12]. Both PEA POD and DD techniques are used in 2C models to derive FFM and FM and are also integral in 3C and 4C models.

Relatively few studies have compared infant body composition derived from PEA POD or DD using either 2C or multi-compartment models. In full-term infants, a 2C model using PEA POD (2C-PP) has shown good agreement with a 2C model using the DD technique (2C-DD) [13] and a 4C model [14], but not with a 2C model using DXA [15]. In pre-term infants, the 2C-PP has shown good agreeability with a 2C model using H_2_^18^O isotope dilution [16] and a 3C model [17]. However, to our knowledge, no study has compared measurements derived from PEA POD and DD techniques using both 2C and 3C models in full-term infants. Moreover, the earlier study [13] to compare PEA POD and DD using a 2C model included predominantly Asian infants. As ethnicity is a significant predictor of infant body composition [18, 19], it may be valuable to test the agreeability of both techniques in infants of other ethnicities.

The overall aim of this study was to appraise the agreeability of body composition measures obtained from PEA POD and DD using 2C and 3C models in 6-month-old infants. Specific aims were to compare FM and %FM derived from (i) 2C-PP vs a 3C model that uses both PEA POD and DD techniques, (ii) 2C-DD vs a 3C model that uses both PEA POD and DD techniques, and (iii) 2C-PP vs 2C-DD.

## Subjects and methods

### Participants

This study was conducted as part of the Baby-bod study [20, 21]—a prospective longitudinal cohort study conducted at the Launceston General Hospital, Tasmania, Australia (September 2017 to October 2019), the Australian arm of the Multicenter Infant Body Composition Reference Study (MIBCRS) [22]. In the Baby-bod study, infants were longitudinally tracked from birth until the age of 6 months. This study utilised data gathered during the 6-month follow-up, incorporating body composition assessments conducted using both the PEA POD and DD techniques. Inclusion criteria of the Baby-bod study were that participating mothers must be 18 years of age or older, able to speak and understand English, have a gestational age at birth between 37^+0^ weeks and 41^+6^ weeks, and have a singleton pregnancy. Exclusion criteria were infants born with congenital birth defects, infants admitted to the neonatal intensive care unit, mothers with significant morbidity, and mothers unable to negotiate the informed consent process due to a difficult birthing experience. All eligible mothers who agreed to their infants’ participation in the study provided signed informed consent.

### Body composition assessment procedures

- Assessment of body density by PEA POD body composition system
  Infant body density was assessed using the PEA POD (COSMED USA, Inc., Concord, CA, USA; software version 3.5.0). The physical design and the operating procedures of the PEA POD have been described in detail elsewhere [13, 23]. In brief, the unclothed infant was first placed on the scale for measuring body mass (M_B_) and then in the test chamber for two minutes for volume measurement (V_B_) to evaluate body density (D_B_ = M_B_/V_B_).
- Assessment of TBW using the DD technique
  Body composition assessment in infants using the DD technique has also been described in detail elsewhere [24]. Briefly, a sample of saliva (pre-dose sample) was collected from the infant who had fasted for 20–30 min using a sterile cotton ball held with sterile plastic forceps. One gram of undiluted deuterium oxide (D_2_O 99.8%) was administered to the infant. Saliva samples were collected at 2.5 hours (post-dose sample I) and 3 hours (post-dose sample II) after the dose administration in the same way described above. D_2_O concentration in each saliva sample was determined using the Agilent 4500 FTIR portable spectroscopy instrument (Agilent Technologies, Inc., USA) [25]. Using the values of concentration (C_1_) and volume (V_1_) of the tracer (D_2_O) in the dose, and the concentration (C_2_) of the tracer in the saliva sample is measured, the volume of distribution (V_2_), also known as dilution space, was calculated using the dilution principle, V_2_ = C_1_V_1_/C_2_. The dilution space is slightly larger than TBW due to the non-aqueous exchange of the isotope. For D_2_O, it is 1.041 times that of TBW; thus, TBW was calculated as V_2_/1.041.

### Estimation of FM and %FM using 2C and 3C models

- 2C model with body density measured by PEA POD
  Whole-body density (D_B_) can be defined as a function of the densities of FM (D_FM_) and FFM (D_FFM_).
  $$\frac{1}{{{\rm{D}}}_{{\rm{B}}}}=\frac{{P}_{{FM}}}{{{\rm{D}}}_{{\rm{FM}}}}+\frac{{{\rm{P}}}_{{FFM}}}{{{\rm{D}}}_{{\rm{FFM}}}}$$
  The above equation can be rearranged as below to express %FM.
  $$\% {\rm{FM}}=\left[\frac{{{{\rm{D}}}_{{\rm{FM}}}{\rm{D}}}_{{\rm{FFM}}}}{{{\rm{D}}}_{{\rm{B}}}\left({{{\rm{D}}}_{{\rm{FFM}}}-{\rm{D}}}_{{\rm{FM}}}\right)}-\frac{{{\rm{D}}}_{{\rm{FM}}}}{\left({{\rm{D}}}_{{\rm{FFM}}}-{{\rm{D}}}_{{\rm{FM}}}\right)}\right]* 100 \%$$
  The density of FM is equal to 0.9007 kg/L and considered to be constant throughout life. Age- and sex-specific estimates for D_FFM_ were derived from the Fomon model [5]. FM and FFM were calculated as below.
  $${\rm{FM}}=\frac{( \% {\rm{FM}}){{\rm{M}}}_{{\rm{B}}}}{100 \% }$$
  $${\rm{FFM}}={{\rm{M}}}_{{\rm{B}}}-{\rm{FM}}$$
- 2C model with TBW assessed by DD technique
  FFM was estimated using the estimated TBW from the DD technique and hydration factor of infants from Fomon et al. [5].
  $${\rm{FFM}}=\frac{{\rm{TBW}}}{{\rm{hydration}}\,{\rm{factor}}}$$
  After estimating FFM, FM and %FM were calculated using the below equations.
  $${\rm{FM}}={{\rm{M}}}_{{\rm{B}}}-{\rm{FFM}}$$
  $$\% \,{\rm{FM}}=\frac{{\rm{FM}}\,(100 \% )}{{{\rm{M}}}_{{\rm{B}}}}$$
- 3C model with body density and TBW

The 3C model divides body mass into FM, TBW, and FFDM, i.e., proteins and minerals. The whole-body densitometry equation can be arranged as below to express the % values of FM and TBW.

$$\frac{100}{{{\rm{D}}}_{{\rm{B}}}}=\frac{ \% {\rm{FM}}}{{{\rm{D}}}_{{\rm{FM}}}}+\frac{ \% {\rm{TBW}}}{{{\rm{D}}}_{{\rm{TBW}}}}+\frac{100- \% {\rm{FM}}- \% {\rm{TBW}}}{{{\rm{D}}}_{{\rm{FFDM}}}}$$

D_FFDM_ was estimated to be 1.4898 kg/L for boys and 1.4909 kg/L for girls by applying the densitometric principle on FFDM as a mixture of proteins and minerals, using densities [26] and sex-specific proportions [5] of proteins and minerals in 6 month-old infants. These values and body density (D_B_) estimated from ADP and percent TBW (%TBW) estimated from DD technique with the assumptions of D_FM_ to be 0.9007 kg/L, D_TBW_ to be 0.9937 kg/L were used to calculate %FM and, thereby, FM.

### Statistical analysis

All analyses were performed using R Project for Statistical Computing (version 4.2.0, Vienna, Austria) [27]. Descriptive variables are expressed using mean and standard deviation (SD). Absolute values of FM in grams and %FM derived by different methods were compared using paired *t*-tests. The degree of agreement between the measures was assessed with Bland-Altman analysis. The expected limits of agreement (LOA) were defined as a priori based on measurements with the 4C reference model [14]. Deming regression analysis [28] was performed to determine systematic bias (constant and proportional bias) between the methods, as it accounts for random errors in both X and Y variables. In an effort to gain a better understanding and clarify the potential factors contributing to the difference in FM and %FM calculated by each method, the differences were plotted against body mass, length, body volume, body density, and %TBW, and a regression line was added to determine the significance of any relationship observed (Supplementary Figs 1–3). All hypothesis tests were two-sided, and p < 0.05 was considered statistically significant.

## Results

Sixty-eight infants (36 boys and 32 girls) were assessed using PEA POD and DD techniques. They were predominantly Caucasian (93%), with a mean age of 5.82 months, mean body mass of 7.25 kg, and mean length of 65.33 cm (Table 1). Within the study cohort, 15% of the infants were exclusively breastfed, while approximately 60% of them relied on breastmilk for over 75% of their dietary intake. Paired *t*-tests revealed no significant differences (p > 0.6 for all) between the means of body composition estimates (FFM, FM and %FM) obtained from different methods (Table 2). On average, their %FM was ~25% in all 3 methods.

**Table 1:** Demographic characteristics of the infants.

Characteristic	Mean (SD)/ n (%)
Gestational age (weeks)	39.70 (1.10)
Age (months)	5.82 (0.30)
Body weight (kg)	7.25 (0.70)
Length (cm)	65.33 (2.21)
Body mass index (kg/m^2^)	16.93 (1.15)
Head circumference (cm)	42.79 (1.28)
Sex*	
Girls	32 (47%)
Boys	36 (53%)
Ethnicity*	
Caucasian	63 (93%)
Other	5 (7%)
Infant feeding at 6 months*^‡^	
Exclusively breastmilk	10 (15%)
>75% of the diet is breastmilk	29 (43%)
>50% of the diet is breastmilk	40 (59%)
No breastmilk	22 (32%)

N = 68; *SD* standard deviation; values for variables marked with * are number of infants (percentage); Infant feeding at six months was assessed using a 24-hour feed recall of the day prior to the assessment; the relative amount of breastmilk included in an infant’s diet was calculated using the following formula [relative amount of breastmilk (%) = (number of breastmilk feeds / total number of feedings per day)×100], and categorised into four groups: “100% of the diet is breastmilk” (exclusively breastmilk), “>75% of the diet is breastmilk”, “>50% of the diet is breastmilk” and “no breastmilk”; ^‡^Percentages do not sum up to 100%.

**Table 2:** Body composition estimates of the infants by different methods.

Parameter	2C-PP	2C-DD	3C
FFM (kg)	5.42 (0.54)	5.42 (0.58)	5.42 (0.54)
FM (kg)	1.84 (0.41)	1.83 (0.49)	1.84 (0.43)
%FM (%)	25.2 (4.5)	25.1 (5.6)	25.2 (4.6)
Body volume (L)	7.12 (0.71)	_	_
Body density (kg/L)	1.019 (0.008)	_	_
TBW (kg)	_	4.32 (0.46)	_
%TBW (%)	_	59.6 (4.5)	_

All values are mean (standard deviation); *FM* fat mass, %*FM* percent fat mass, *TBW* total body water, %*TBW* percent total body water, *2C-PP* two-compartment model with PEA POD, *2C-DD* two-compartment model with deuterium dilution technique, *3C* three-compartment model, no significant differences in FFM, FM or %FM between the methods (p > 0.6 for all).

**Fig. 1:**
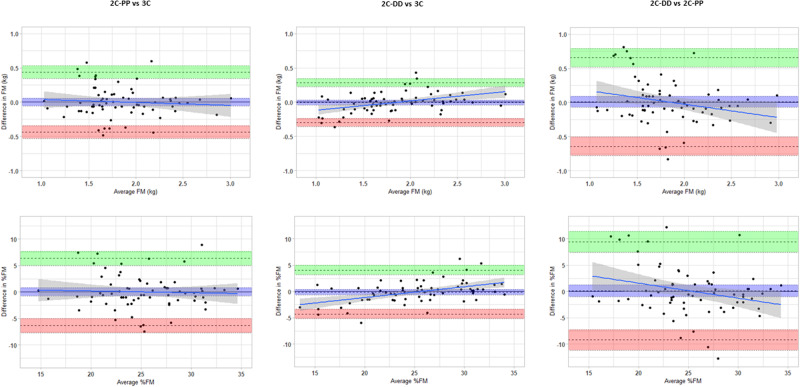
Bland–Altman analyses comparing the two-compartment model with PEA POD to the three-compartment model (2C-PP vs 3C), the two-compartment model with deuterium dilution to the three-compartment model (2C-DD vs 3C), and the two-compartment model with deuterium dilution to the two-compartment model with PEA POD (2C-DD vs 2C-PP). The panels in the top row display Bland–Altman analyses of fat mass (FM) for 2C-PP vs 3C (top left), 2C-DD vs 3C (top middle) and 2C-DD vs 2C-PP (top right). The panels in the bottom row display Bland–Altman analyses of percent fat mass (%FM) for 2C-PP vs 3C (bottom left), 2C-DD vs 3C (bottom middle) and 2C-DD vs 2C-PP (bottom right). In each panel, the *Y* axes show the difference between the methods, and the *X* axes show the mean of the methods pertaining to the respective body composition variable. The solid black line represents the mean differences between the methods, and the dashed lines are the limits of agreement (±2 SD from the mean difference). The blue colour diagonal line represents the proportional bias; coloured shaded areas around solid and dashed lines show 95% confidence intervals.

Bland-Altman results showed that the mean differences in FM and %FM between methods (mean bias) were not significant and close to zero (Table 3, Fig. 1). The LOA were widest in the comparison of 2C-PP vs 2C-DD (Lower LOA and Upper LOA for FM: -0.643, 0.657; for %FM: -9.217, 9.436), followed by 2C-PP vs 3C (FM: –0.439, 0.439; %FM: –6.352 to 6.352) and it was comparatively narrower in 2C-DD vs 3C comparison (FM: -0.296, 0.282; %FM: –4.278, 4.061). No trends were observed in the scatter of the points on the plots of 2C-PP vs 3C (slopes were not significant). Positive trends were evident along the graphs of 2C-DD vs 3C, with 2C-DD underestimating infant fatness compared to 3C at lower mean values and overestimating at higher mean values. In contrast, negative trends were observable in the plots of 2C-PP vs 2C-DD, with 2C-PP overestimating infants’ fatness compared to 2C-DD at lower mean values and underestimating it at higher mean values.

**Table 3:** Estimates of Bland-Altman analyses.

Parameter	2C-PP vs 3C	2C-DD vs 3C	2C-PP vs 2C-DD
FM			
Mean difference	–0.0001 (–0.054, 0.054)	–0.007 (–0.043, 0.029)	0.007 (–0.073, 0.087)
Upper LOA	0.439 (0.346, 0.533)	0.282 (0.221, 0.344)	0.657 (0.519, 0.7949)
Lower LOA	–0.439 (–0.533, –0.346)	–0.296 (–0.358, –0.235)	-0.643 (-0.789, -0.505)
Slope	–0.043 (–0.178, 0.093)	0.135 (0.062, 0.207)	-0.196 (-0.384, -0.009)
%FM			
Mean difference	0.0003 (–0.784, 0.785)	–0.109 (–0.624, 0.406)	0.109 (-1.043, 1.261)
Upper LOA	6.352 (5.005, 7.699)	4.061 (3.176, 4.945)	9.436 (7.458, 11.414)
Lower LOA	–6.352 (–7.699, –5.005)	–4.278 (–5.163, –3.394)	-9.217 (-11.196, -7.239)
Slope	–0.032 (–0.220, 0.156)	0.205 (0.114, 0.297)	-0.287 (-0.539, -0.035)

All values are estimate (95% confidence interval); *FM* fat mass, %*FM* percent fat mass, *2C-PP* two-compartment model with PEA POD, *2C-DD* two-compartment model with deuterium dilution technique, *3C* three-compartment model, *LOA* limits of agreement.

**Fig. 2:**
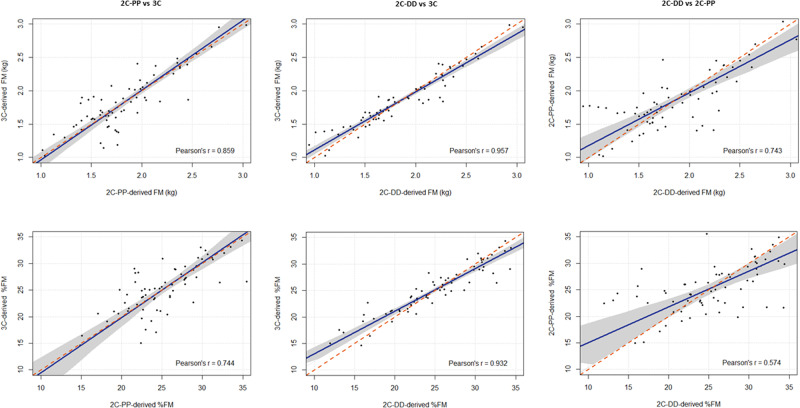
Deming regression analyses comparing the two-compartment model with PEA POD to the three-compartment model (2C-PP vs 3C), the two-compartment model with deuterium dilution to the three-compartment model (2C-DD vs 3C), and the two-compartment model with deuterium dilution to the two-compartment model with PEA POD (2CDD vs 2C-PP). The panels in the top row display Deming regression analyses of fat mass (FM) for 2C-PP vs 3C (top left), 2C-DD vs 3C (top middle) and 2C-DD vs 2C-PP (top right). The panels in the bottom row display Deming regression analyses of percent fat mass (%FM) for 2C-PP vs 3C (bottom left), 2C-DD vs 3C (bottom middle) and 2C-DD vs 2C-PP (bottom right). In each panel, the regression line is given in ‘blue’, and the line of identity (*Y* = *X*) is given in ‘red’; Shaded areas show 95% confidence interval for the regression line; the Pearson correlation coefficient is shown in each panel.

Deming regression analysis revealed no constant or proportional differences and no major departure from the line of identity in the comparison of 2C-PP vs 3C. However, in the comparisons of 2C-DD vs 3C and 2C-PP vs 2C-DD, the regression line deviated from 1, and constant and proportional differences were observed (Table 4, Fig. 2). Additionally, the mean differences in FM and %FM derived from the different methods comparisons (2C-PP vs 3C, 2C-DD vs 3C and 2C-PP vs 2C-DD) were significantly associated with %TBW (p < 0.001) but not with body mass, length, or volume (p > 0.4 for all; Supplementary Figs 1–3). Body density was significantly associated with the mean differences in 2C-PP vs 3C and 2C-PP vs 2C-DD (p < 0.05), but not in 2C-DD vs 3C (p > 0.4).

**Table 4:** Estimates of Deming regression analyses.

Parameter	2C-PP vs 3C	2C-DD vs 3C	2C-PP vs 2C-DD
FM			
Intercept	-0.087 (-0.396, 0.119)	0.243 (0.109, 0.370)	0.383 (0.032, 0.725)
Slope	1.047 (0.939, 1.199)	0.871 (0.803, 0.940)	0.794 (0.616, 0.964)
%FM			
Intercept	-0.970 (-7.457, 3.959)	4.933 (2.528, 6.855)	8.209 (1.275, 14.424)
Slope	1.038 (0.846, 1.290)	0.808 (0.733, 0.897)	0.677 (0.433, 0.943)

All values are estimate (95% confidence interval); *FM* fat mass, %*FM* percent fat mass, *2C-PP* two-compartment model with PEA POD, *2C-DD* two-compartment model with deuterium dilution technique, *3C* three-compartment model.

## Discussion

In this study, we investigated the agreement of body composition measures obtained from PEA POD and DD techniques using 2C and 3C models in 6-month-old infants. Mean differences between the methods were not significant. However, significant constant and proportional bias were noted in comparisons of 2C-DD vs 3C and 2C-DD vs 2C-PP, but not in 2C-PP vs 3C comparison. In addition, significant associations were observed between the mean differences and %TBW.

Our findings may be particularly relevant in clinical settings where precise assessments of infant body composition are fundamental for growth monitoring and nutritional management. The absence of significant mean differences among the methods is an encouraging finding for researchers and healthcare professionals engaged in infant health assessments, as they can confidently choose from these methods, knowing that, on average, they provide similar body composition estimates. However, it is essential to acknowledge methodological nuances, including varying LOA, especially notable in the 2C-PP vs 2C-DD comparison, and potential systematic variations, particularly the constant and proportional biases observable in the 2C-DD vs 3C and 2C-DD vs 2C-PP comparisons. These differences suggest potential discrepancies in individual measurements, particularly in infants whose body fat levels deviate significantly from the typical range, either being extremely below or above the average. Further, our study revealed significant associations between mean differences in FM and %FM and %TBW, inferring variations in %TBW may influence the results obtained from different methods. This emphasises the importance of adopting a holistic approach for assessing infant body composition that considers not only methodological choices but also close control of pre-analytical factors, such as the hydration status of the participants.

Studies that determined the agreeability of body composition values derived from PEA POD and DD using 2C models, either with each other or with other techniques that used 2C or multi-compartment models, have been limited. Ma et al. [13] reported an excellent agreement between %FM assessed with 2C-PP and 2C-DD with identical mean values, and similarly, in our study, the mean difference in %FM did not differ significantly from zero. However, Ma et al. did not observe any proportional bias as we observed in our study, and their LOA for %FM (-6.84%, 6.71%) was narrower than ours (-9.22%, 9.44%). There could be several potential reasons for these discrepancies. Firstly, infants in the present study were older (5.82 months vs 1.34 months, respectively) and predominantly Caucasian vs Asian in the earlier study. Asian infants are characterised by higher FM and lower FFM than white Caucasians [29, 30]. We performed the analysis excluding the infants from ethnicities other than Caucasian (n = 5) and found no significant differences between the results. Secondly, as shown in supplementary figures, the significant variation in %TBW of infants in our study may have influenced the results; principles used in PEA POD are based on assumptions of body density, ignoring the inter-individual variation in the constituents of FFM. We also observed that the associations between body density and mean differences were only significant when comparing 2C-PP, which does not consider inter-individual variations in %TBW, with other methods.

Kuriyan et al. [31] evaluated the agreement of body composition estimates from 2C-PP and 2C-DD using pooled data from the larger Multicenter Infant Body Composition Reference Study (MIBCRS), which involved infants from Australia (n = 46), India (n = 86), and South Africa (n = 44), and reported a low mean difference (-0.05 kg; p < 0.05) in pooled FM derived from the two methods. However, the results of the present study showed no significant mean differences in FM in Australian infants (n = 66), suggesting the differences in the former study [31] may have arisen from different numbers and ethnicities of participating infants. Moreover, Ellis et al. [14] compared %FM in full-term infants (age 2–17 weeks) derived by 2C-PP against a 4C and reported no significant mean bias. In their study, LOA was wider (-6.8% to 8.1%) than the study by Ma et al. [13] but narrower than ours. Further, a study by Fields et al. [15] compared FM and %FM using 2C-PP and 2C-DXA in term-born infants at 6 months of age and reported that estimates of FM (2.284 vs 1.921 kg; p < 0.001) and %FM (31.1% vs 26.7%; p < 0.001) by 2C-DXA were significantly greater than those by 2C-PP. They showed that the difference in %FM reduced with increasing mean %FM values, and a significant association existed between body mass and infant fatness; specifically, significant differences in FM and %FM occurred when infant body mass was less than 7 kg. In contrast, the mean differences in our study were not dependent on infants’ body mass, length, or volume.

A 4C model to independently assess FFM constituents is considered the gold standard for body composition assessment. In contrast, 2C models are based on several assumptions [8] and may not provide the most accurate estimations of body composition [32]. Fields et al. [33] compared the accuracy of FM assessed by ADP (BOD POD), DD, DXA, and hydrostatic weighing against a 4C model and concluded that ADP prevailed over the other methods. They also highlighted that DD-2C might be associated with the highest error as it assumes a constant hydration status, but individuals may have significant differences in hydration status. Our results support this finding, as there was no constant or proportional bias between the estimates derived from PEA POD with a 2C model and a 3C model, whereas significant proportional bias existed when the DD technique was used in a 2C model. Other plausible reasons for the lower accuracy of the 2C-DD may include dose spillages [12], although we took all precautions to ensure that infants consumed the doses completely. Insensible water loss during the equilibration period may be another issue [24]; we assumed that the loss of deuterium in urine and sweat was minimal and could be ignored.

PEA POD software assigns values for the density of FFM based on the age and sex of the infant. Additionally, to account for differences in the compressibility of air in the thoracic cavity and near the skin surface, the PEA POD software makes corrections in the measured body volume by predicting thoracic gas volume and surface area artefacts [23]. These predictions may introduce errors at the individual level. Moreover, hair, body moisture and temperature have been shown to significantly affect %FM measurements by ADP, with increases in body temperature and moisture resulting in underestimation of body fatness [34, 35]. Despite these inherent potential sources of errors, the PEA POD is widely considered a reliable and valid tool to track body composition during the postnatal period, as less erroneous multi-compartment models are expensive and especially impractical with infants [8, 13, 14, 36].

Ours is the first study to compare PEA POD and DD measurements using 2C and 3C models in term-born, predominantly Caucasian infants. Our study has a reasonable sample size compared to similar studies [13–15], and participating infants were all approximately the same age (6 months), limiting the effect age-related variations in body composition may have on comparisons. Due to the practical challenges of working with infants and for reducing participant burden, we did not perform repeated body composition assessments. Consequently, we could not calculate within-day and between-day coefficients of variations (CVs) for body composition measurements, which would have provided insights into the precision and stability of the body composition measurements. Moreover, a comparison with a “gold standard” 4C model would have provided further information on the accuracy of the results.

In conclusion, while we found no significant mean differences in body composition estimates between the methods, it is noteworthy that we identified significant constant and proportional biases in specific comparisons. Specifically, biases were observed when comparing 2C-DD to both 3C and 2C-PP methods, but no such biases were evident in the 2C-PP vs 3C comparison. The observed proportional bias suggests that applying these methods to infants with body fat levels below or above the average can result in substantial differences in the estimates. In addition, the significant associations between the mean differences and %TBW emphasise that the hydration state of the participants can influence the accuracy and reliability of the results. These results underline the need for careful consideration of the choice of method, the importance of maintaining methodological consistency in longitudinal studies, and the need to consider and control various pre-analytical factors, such as the hydration state of the participants. On the whole, our findings contribute valuable insights to the intricate field of infant body composition assessment, encouraging further investigations to elucidate sources of bias and continued research and refinement of measurement techniques to enhance accuracy and reliability in this challenging population.

## Supplementary information

Supplementary Figures

## Data Availability

Data are available upon request from authors.

## References

[1] Yajnik CS. Transmission of obesity-adiposity and related disorders from the mother to the baby. Ann Nutr Metab. 2014;64 Suppl 1:8–17. 25059801 10.1159/000362608

[2] Ratnasingham A, Eiby YA, Nitert MD, Donovan T, Lingwood BE. Review: Is rapid fat accumulation in early life associated with adverse later health outcomes? Placenta. 2017;54:125–30. 28104278 10.1016/j.placenta.2017.01.101

[3] Ay L, Hokken-Koelega AC, Mook-Kanamori DO, Hofman A, Moll HA, Mackenbach JP. Tracking and determinants of subcutaneous fat mass in early childhood: the generation R study. Int J Obes. 2008;32:1050–9. 10.1038/ijo.2008.76 18560371

[4] Duren DL, Sherwood RJ, Czerwinski SA, Lee M, Choh AC, Siervogel RM, et al. Body composition methods: comparisons and interpretation. J Diabetes Sci Technol. 2008;2(6):1139–46. 19885303 10.1177/193229680800200623 PMC2769821

[5] Fomon SJ, Haschke F, Ziegler EE, Nelson SE. Body composition of reference children from birth to age 10 years. Am J Clin Nutr. 1982;35:1169–75. 7081099 10.1093/ajcn/35.5.1169

[6] Wells JC, Williams JE, Ward LC, Fewtrell MS. Utility of specific bioelectrical impedance vector analysis for the assessment of body composition in children. Clin Nutr. 2021;40:1147–54. 32788087 10.1016/j.clnu.2020.07.022 PMC7957362

[7] Wells JC, Fewtrell MS. Measuring body composition. Arch Dis Child. 2006;91:612–7. 16790722 10.1136/adc.2005.085522 PMC2082845

[8] Demerath EW, Fields DA. Body composition assessment in the infant. Am J Hum Biol. 2014;26:291–304. 24424686 10.1002/ajhb.22500 PMC5761669

[9] Fields DA, Gunatilake R, Kalaitzoglou E. Air displacement plethysmography: cradle to grave. Nutr Clin Pract. 2015;30:219–26. 25761768 10.1177/0884533615572443

[10] Lee SY, Gallagher D. Assessment methods in human body composition. Curr Opin Clin Nutr Metab Care. 2008;11:566–72. 18685451 10.1097/MCO.0b013e32830b5f23 PMC2741386

[11] Fields DA, Allison DB. Air-displacement plethysmography pediatric option in 2-6 years old using the four-compartment model as a criterion method. Obesity (Silver Spring, Md). 2012;20:1732–7. 22421895 10.1038/oby.2012.28 PMC3628559

[12] Nielsen SB, Wells JC, Slater C, Fewtrell MS, Reilly JJ. Administering labelled water to exclusively breast-fed infants in studies involving stable isotope dilution techniques. Isotopes Environ Health Stud. 2011;47:18–25. 21390987 10.1080/10256016.2011.554980

[13] Ma G, Yao M, Liu Y, Lin A, Zou H, Urlando A, et al. Validation of a new pediatric air-displacement plethysmograph for assessing body composition in infants. Am J Clin Nutr. 2004;79:653–60. 15051611 10.1093/ajcn/79.4.653

[14] Ellis KJ, Yao M, Shypailo RJ, Urlando A, Wong WW, Heird WC. Body-composition assessment in infancy: air-displacement plethysmography compared with a reference 4-compartment model. Am J Clin Nutr. 2007;85:90–5. 17209182 10.1093/ajcn/85.1.90

[15] Fields DA, Demerath EW, Pietrobelli A, Chandler-Laney PC. Body composition at 6 months of life: comparison of air displacement plethysmography and dual-energy X-ray absorptiometry. Obesity (Silver Spring). 2012;20:2302–6. 22522885 10.1038/oby.2012.102

[16] Roggero P, Gianni ML, Amato O, Piemontese P, Morniroli D, Wong WW. Evaluation of air-displacement plethysmography for body composition assessment in preterm infants. Pediatr Res. 2012;72:316–20. 22669294 10.1038/pr.2012.75

[17] Forsum E, Olhager E, Törnqvist C. An evaluation of the pea pod system for assessing body composition of moderately premature infants. Nutrients. 2016;8:238. 27110820 10.3390/nu8040238 PMC4848706

[18] Koo WWK, Walters JC, Hockman EM. Body composition in human infants at birth and postnatally. J Nutr. 2000;130:2188–94. 10958811 10.1093/jn/130.9.2188

[19] Sauder KA, Kaar JL, Starling AP, Ringham BM, Glueck DH, Dabelea D. Predictors of infant body composition at 5 months of age: the healthy start study. J Pediatr. 2017;183:94–9.e1. 28161200 10.1016/j.jpeds.2017.01.014 PMC5367947

[20] Herath MP, Ahuja KDK, Beckett JM, Jayasinghe S, Byrne NM, Hills AP. Determinants of infant adiposity across the first 6 months of life: evidence from the baby-bod study. J Clin Med. 2021;10:1770. 33921680 10.3390/jcm10081770 PMC8073882

[21] Jayasinghe S, Herath MP, Beckett JM, Ahuja KDK, Byrne NM, Hills AP. WHO Child Growth Standards in context: The Baby–bod Project - Observational study in Tasmania. BMJ Paediatr Open. 2021;5:e001123. 34222680 10.1136/bmjpo-2021-001123 PMC8211047

[22] Murphy-Alford AJ, Johnson W, Nyati LH, Santos IS, Hills AP, Ariff S, et al. Body composition reference charts for infants from birth to 24 months: Multicenter Infant Body Composition Reference Study. Am J Clin Nutr. 2023;117:1262–9. 37270290 10.1016/j.ajcnut.2023.02.012

[23] Urlando A, Dempster P, Aitkens S. A new air displacement plethysmograph for the measurement of body composition in infants. Pediatr Res. 2003;53:486–92. 12595599 10.1203/01.PDR.0000049669.74793.E3

[24] International Atomic Energy Agency. Introduction to body composition assessment using the deuterium dilution technique with analysis of saliva samples by Fourier transform infrared spectrometry. Vienna: International Atomic Energy Agency; 2010.

[25] International Atomic Energy Agency. Stable Isotope Technique to Assess Intake of Human Milk in Breastfed Infants. Vienna: IAEA Human Health Series No. 7; 2010.

[26] Brozek J, Grande F, Anderson JT, Keys A. Densitometric analysis of body composition: revision of some quantitative assumptions. Ann NY Acad Sci. 1963;110:113–40. 14062375 10.1111/j.1749-6632.1963.tb17079.x

[27] R Core Team. R: A Language and Environment for Statistical Computing. In: R Foundation for Statistical Computing, editor. 2020.

[28] Martin RF. General deming regression for estimating systematic bias and its confidence interval in method-comparison studies. Clin Chem. 2000;46:100–4. 10620577

[29] Stanfield KM, Wells JC, Fewtrell MS, Frost C, Leon DA. Differences in body composition between infants of South Asian and European ancestry: the London Mother and Baby Study. 2012;41:1409-18. 10.1093/ije/dys139 PMC3465771 22984147

[30] Anand SS, Gupta MK, Schulze KM, Desai D, Abdalla N, Wahi G, et al. What accounts for ethnic differences in newborn skinfold thickness comparing South Asians and White Caucasians? Findings from the START and FAMILY Birth Cohorts. Int J Obes. 2016;40:239–44. 10.1038/ijo.2015.171 PMC4753357 26315840

[31] Kuriyan R, Hills A, Murphy-Alford A, Padmanabha R, Nyati L, Byrne N, et al. Body composition of infants at 6 months of age using a 3-compartment model. 2023:PREPRINT (Version 1) available at Research Square 10.21203/rs.3.rs-2798935/v1. 10.1038/s41430-023-01351-2 PMC11537952 37833566

[32] Lohman TG. Applicability of body composition techniques and constants for children and youths. Exerc Sport Sci Rev. 1986;14:325–57. 3525188

[33] Fields DA, Goran MI. Body composition techniques and the four-compartment model in children. J Appl Physiol. 2000;89:613–20. 10926645 10.1152/jappl.2000.89.2.613

[34] Higgins PB, Fields DA, Hunter GR, Gower BA. Effect of scalp and facial hair on air displacement plethysmography estimates of percentage of body fat. Obes Res. 2001;9:326–30. 11346675 10.1038/oby.2001.41

[35] Fields DA, Higgins PB, Hunter GR. Assessment of body composition by air-displacement plethysmography: influence of body temperature and moisture. Dyn Med. 2004;3:3. 15059287 10.1186/1476-5918-3-3 PMC411054

[36] Mazahery H, von Hurst PR, McKinlay CJD, Cormack BE, Conlon CA. Air displacement plethysmography (pea pod) in full-term and pre-term infants: a comprehensive review of accuracy, reproducibility, and practical challenges. Maternal Health Neonatol Perinatol. 2018;4:12. 10.1186/s40748-018-0079-z PMC6011189 29951209

