# Supplementary material for: Body composition assessment in 6-month-old infants: A comparison of two- and three-compartment models using data from the Baby-bod study
Source: Eur J Clin Nutr. 2024 Jan 17;78(11):963–9. doi: 10.1038/s41430-023-01394-5 (PMC11537955; doi:10.1038/s41430-023-01394-5)
Supplement: Supplementary file 1 — Supplementary Figures [file 41430_2023_1394_MOESM1_ESM.docx]

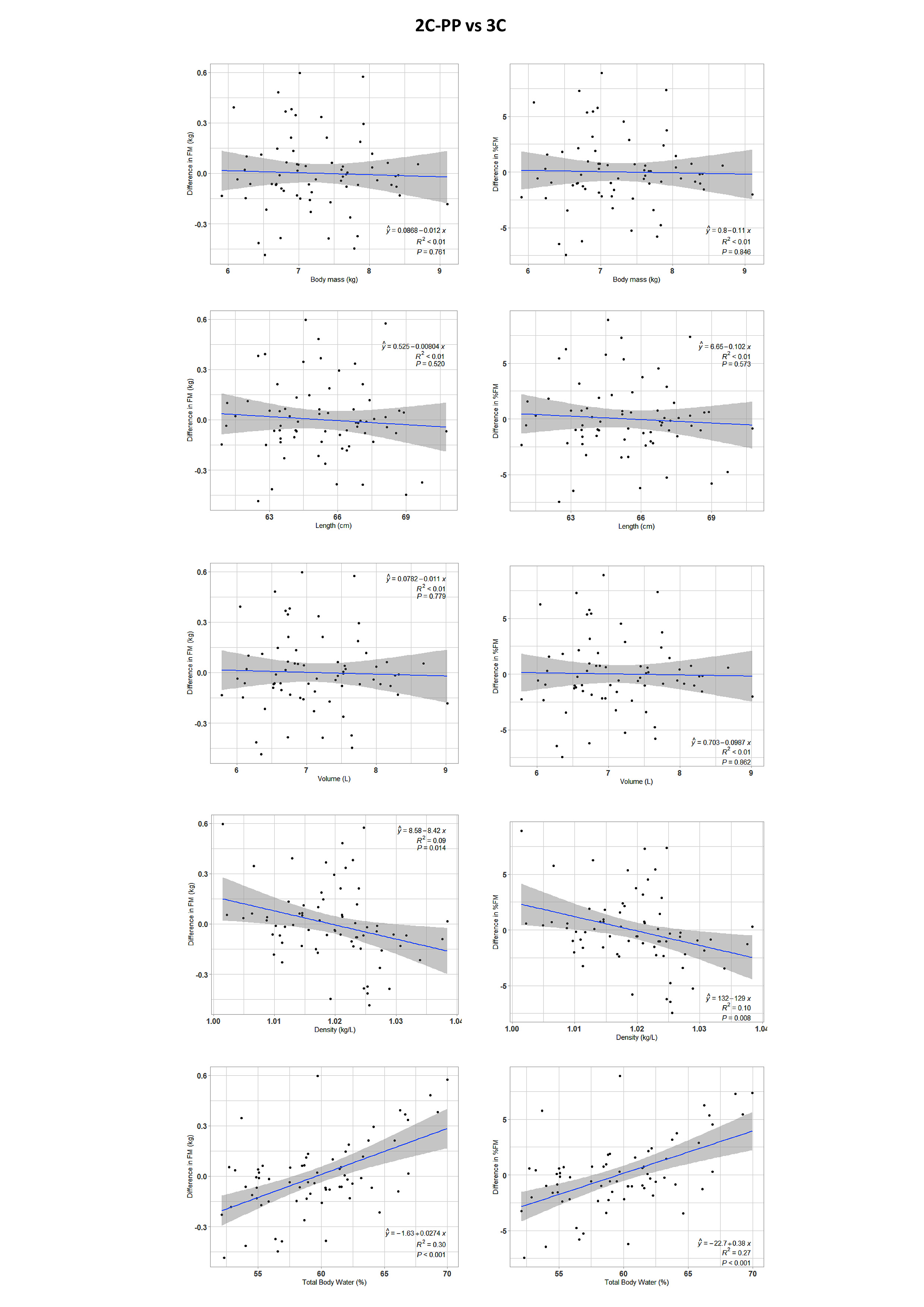


Supplementary figure 1: Linear regressions of differences in fat mass (FM) and percent fat mass (%FM) by PEA POD as a 2-compartment model (2C-PP) vs 3-compartment model (3C) on infants’ body mass, length, volume, density and total body water (%). Regression line is given in ‘blue’ and shaded areas show 95% confidence interval for regression line; Regression equations, coefficient of determination (R^2^) and p-value are shown in each plot.


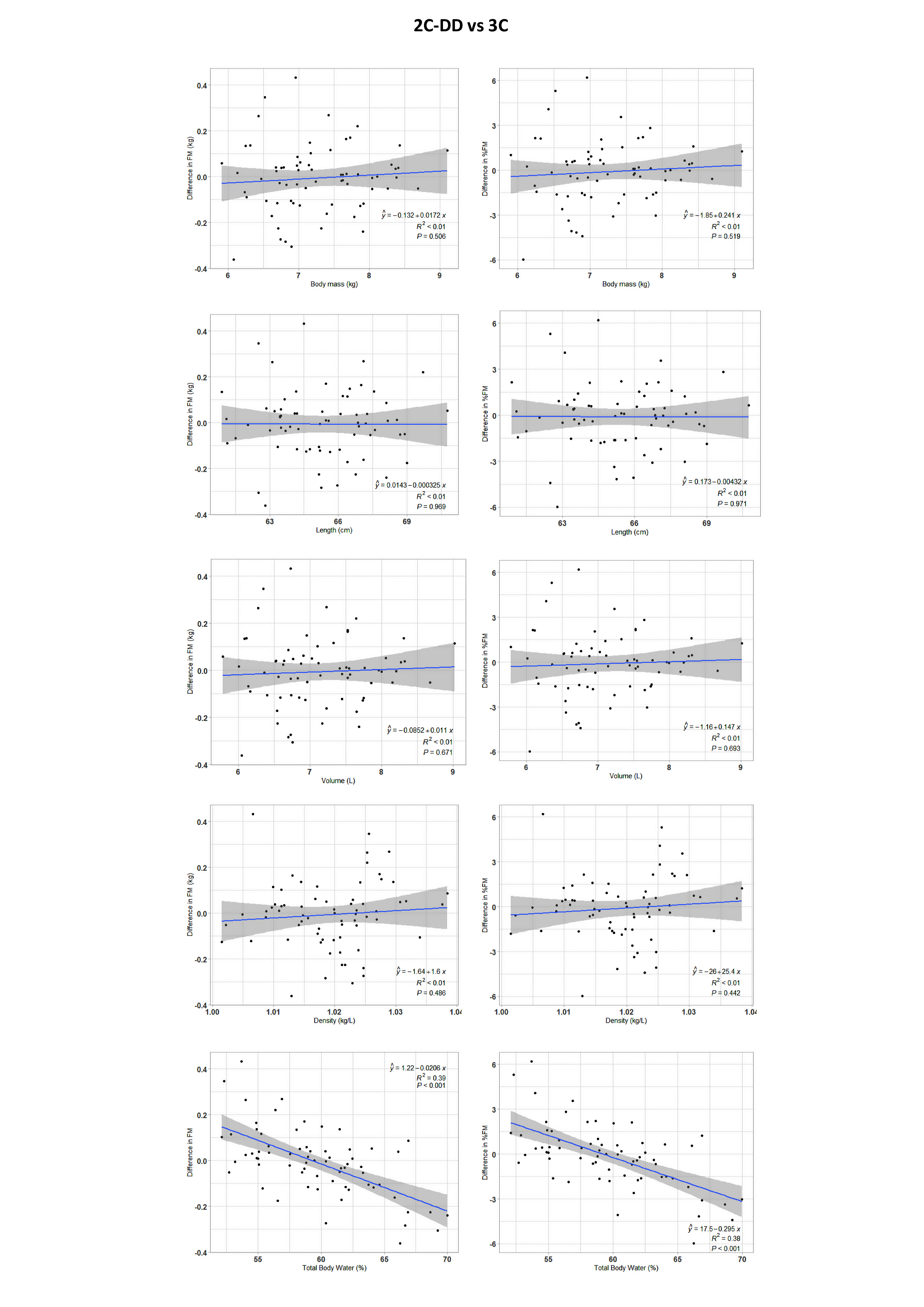


Supplementary figure 2: Linear regressions of differences in fat mass (FM) and percent fat mass (%FM) by deuterium dilution as a 2-compartment model (2C-DD) vs 3-compartment model (3C) on infants’ body mass, length, volume, density and total body water (%). Regression line is given in ‘blue’ and shaded areas show 95% confidence interval for regression line; Regression equations, coefficient of determination (R^2^) and p-value are shown in each plot.


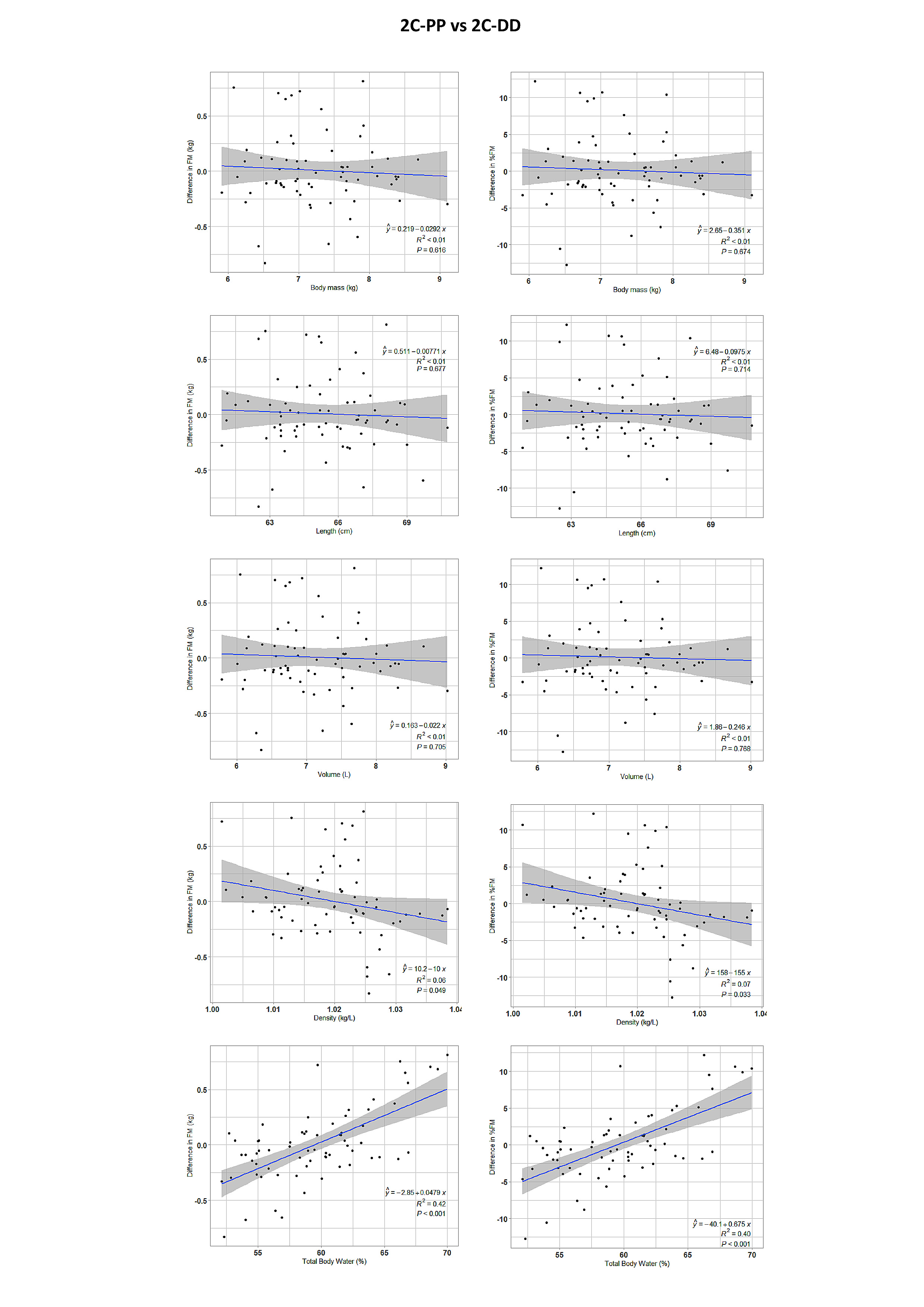


Supplementary figure 3: Linear regressions of differences in fat mass (FM) and percent fat mass (%FM) by PEA POD as a 2-compartment model (2C-PP) vs deuterium dilution as a 2-compartment model (2C-DD) on infants’ body mass, length, volume, density and total body water (%). Regression line is given in ‘blue’ and shaded areas show 95% confidence interval for regression line; Regression equations, coefficient of determination (R^2^) and p-value are shown in each plot.
